# Factors associated with false‐negative cardiovascular magnetic resonance perfusion studies: A Clinical evaluation of magnetic resonance imaging in coronary artery disease (CE‐MARC) substudy

**DOI:** 10.1002/jmri.25032

**Published:** 2015-08-18

**Authors:** Ananth Kidambi, Steven Sourbron, Neil Maredia, Manish Motwani, Julia M. Brown, Jane Nixon, Colin C. Everett, Sven Plein, John P. Greenwood

**Affiliations:** ^1^ Multidisciplinary Cardiovascular Research Centre & the Division of Cardiovascular and Diabetes ResearchLeeds Institute of Cardiovascular and Metabolic Medicine, University of LeedsLeedsUK; ^2^ Division of Medical PhysicsUniversity of LeedsLeedsUK; ^3^ Clinical Trials Research UnitUniversity of LeedsLeedsUK

**Keywords:** magnetic resonance imaging, cardiovascular magnetic resonance, ischemia, coronary disease, perfusion

## Abstract

**Purpose:**

To examine factors associated with false‐negative cardiovascular magnetic resonance (MR) perfusion studies within the large prospective Clinical Evaluation of MR imaging in Coronary artery disease (CE‐MARC) study population. Myocardial perfusion MR has excellent diagnostic accuracy to detect coronary heart disease (CHD). However, causes of false‐negative MR perfusion studies are not well understood.

**Materials and Methods:**

CE‐MARC prospectively recruited patients with suspected CHD and mandated MR, myocardial perfusion scintigraphy, and invasive angiography. This subanalysis identified all patients with significant coronary stenosis by quantitative coronary angiography (QCA) and MR perfusion (1.5T, *T*_1_‐weighted gradient echo), using the original blinded image read. We explored patient and imaging characteristics related to false‐negative or true‐positive MR perfusion results, with reference to QCA. Multivariate regression analysis assessed the likelihood of false‐negative MR perfusion according to four characteristics: poor image quality, triple‐vessel disease, inadequate hemodynamic response to adenosine, and Duke jeopardy score (angiographic myocardium‐at‐risk score).

**Results:**

In all, 265 (39%) patients had significant angiographic disease (mean age 62, 79% male). Thirty‐five (5%) had false‐negative and 230 (34%) true‐positive MR perfusion. Poor MR perfusion image quality, triple‐vessel disease, and inadequate hemodynamic response were similar between false‐negative and true‐positive groups (odds ratio, OR [95% confidence interval, CI]: 4.1 (0.82–21.0), *P* = 0.09; 1.2 (0.20–7.1), *P* = 0.85, and 1.6 (0.65–3.8), *P* = 0.31, respectively). Mean Duke jeopardy score was significantly lower in the false‐negative group (2.6 ± 1.7 vs. 5.4 ± 3.0, OR 0.34 (0.21–0.53), *P* < 0.0001).

**Conclusion:**

False‐negative cardiovascular MR perfusion studies are uncommon, and more common in patients with lower angiographic myocardium‐at‐risk. In CE‐MARC, poor image quality, triple‐vessel disease, and inadequate hemodynamic response were not significantly associated with false‐negative MR perfusion. J. MAGN. RESON. IMAGING 2016;43:566–573.

Coronary Heart Disease (CHD) is the most frequent cause of death in developed countries.^1^ Myocardial perfusion magnetic resonance imaging (MRI) is increasingly used as an accurate and reproducible technique for noninvasive diagnosis of coronary heart disease (CHD). Its potential advantages include a lack of ionizing radiation exposure, cost‐effectiveness, and additional information on cardiac anatomy, function, and tissue viability when performed as part of a multiparametric protocol.^2^, ^3^ Magnetic resonance (MR) perfusion imaging has high sensitivity and specificity as compared to x‐ray coronary angiography.^4^ Furthermore, a normal MR perfusion scan confers excellent short‐to‐medium term prognosis.^5^

False‐negative perfusion MR studies are an important limitation of the method, reported to occur in ∼16% of cases and potentially denying patients appropriate revascularization treatment.^6^ Reasons for false‐negative perfusion MR studies include suboptimal image quality, technical failure, and the inconsistent relationship between angiographic stenosis severity and myocardial ischemia in studies using angiographic endpoints.^7^ False‐negative MR studies are difficult to identify and evaluate, in part due to the large number of patients required for such an analysis and the lack of a consistent reference standard applied to patients with negative noninvasive imaging, with potential for patient selection bias in clinical populations. The Clinical Evaluation of MAgnetic Resonance imaging in Coronary artery disease (CE‐MARC) study prospectively enrolled 752 patients with suspected CHD, providing a large and homogenous patient population for subanalyses.^8^, ^9^ All patients in CE‐MARC were scheduled to undergo MR and myocardial perfusion scintigraphy using single photon‐emission computed tomography (SPECT) (in a randomized order) plus radiographic coronary angiography,^10^ and the study was notable in having both a large study population and a consistent reference standard applied to all patients, including those with negative noninvasive imaging. The CE‐MARC dataset is potentially suited to explore factors associated with false‐negative MR perfusion results in a large prospective population, and was studied for this purpose.

## Materials and Methods

### Patients

CE‐MARC screened and enrolled 752 consecutive patients with suspected angina pectoris between March 2006 and August 2009. Methods, demographics, and primary outcome analysis for the CE‐MARC study have been published previously.^8^, ^10^ Inclusion criteria to CE‐MARC were at least one major cardiovascular risk factor and suspected stable angina needing investigation, as judged by a cardiologist in accordance with contemporary clinical practice. All patients were scheduled to receive cardiovascular MRI and SPECT (in a randomized order), followed by radiographic coronary angiography within 4 weeks. Exclusion criteria were previous coronary artery bypass surgery; pregnancy; inability to lie supine; glomerular filtration rate ≤30 ml/min/1.73m^2^; contraindication to MR (eg, MR unsafe pacemaker); or adenosine infusion. The study protocol was approved by the institutional Research Ethics Committee and complied with the Declaration of Helsinki; all patients gave written informed consent.

For the current analysis, we included all CE‐MARC patients where the original, blinded read of quantitative coronary angiography (QCA) showed one or more significant coronary stenosis (≥70% stenosis of a major coronary artery ≥2 mm diameter, or left main stem stenosis ≥50%). This subpopulation with angiographically significant CHD was divided into a false‐negative group and a true‐positive group based on the MR perfusion result. The false‐negative group comprised all patients in whom the original, blinded read of the MR perfusion component was graded as normal or probably normal; the true‐positive group comprised all patients who had an abnormal MR perfusion component. MR perfusion studies considered of analyzable quality in the original CE‐MARC read were included in the analysis. Four characteristics were prespecified as candidate factors for false‐negative MR perfusion: poor quality stress perfusion MR images, triple‐vessel disease, inadequate hemodynamic response to stress, and Duke jeopardy score.^11^ Although not part of the prespecified multivariate analysis, we also examined myocardial perfusion reserve (MPR) and SPECT assessment of perfusion in the false‐negative patients, in order to further evaluate the effect of coronary stenosis on myocardial perfusion.

### Imaging Methods

MR was performed at 1.5T (Intera CV, Philips Healthcare, Best, The Netherlands) and included cine imaging, stress and rest perfusion, coronary MR angiography, and late gadolinium enhancement (LGE).

Myocardial perfusion MR was performed during adenosine stress (140 mcg/kg/min for 4 minutes) and at rest, using 0.05 mmol/kg of gadolinium‐DTPA (dimeglumine gadopentetate; Magnevist, Bayer, Berlin, Germany) for each acquisition. A *T*_1_‐weighted saturation‐recovery, single‐shot gradient echo pulse sequence was acquired, in three 10‐mm short‐axis slices, TE/TR 1.0/2.7 msec, 15° flip angle, matrix 144 × 144, 320–460 mm field of view (in‐plane spatial resolution 2.2–3.2 mm), sensitivity encoding (SENSE) factor 2, and a single saturation prepulse per R–R interval.^10^ SPECT gated rest and adenosine stress was performed with a 2‐day protocol, using a dedicated cardiac gamma camera (MEDISO Cardio‐C, Budapest, Hungary). A weight‐adjusted dose of 400–600 MBq of ^99m^Tc tetrofosmin (Myoview) was administered for each study, with acquisition of eight gated frames per cardiac cycle with a matrix size of 64 × 64 were acquired. Transaxial stress and rest slices of 6 mm thickness (spatial resolution of ∼10 mm) were reconstructed with a Butterworth scattered back‐projection filter, cutoff frequency of 0.4 Nyquist, and an order of 6 without use of attenuation correction, and reorientated to the cardiac axes for analysis. Adenosine stress perfusion was performed using a similar protocol to MR, with isotope injection after at least 4 minutes of 140 μg/kg/min intravenous adenosine. Invasive x‐ray coronary angiography was carried out using standard clinical methods.^8^, ^10^

### Image Interpretation

All CE‐MARC data were analyzed by consensus of blinded, paired readers with at least 10 years of experience of their imaging modality and blinded to the results of all other modalities. The original CE‐MARC read was used for this substudy; no further image review was undertaken to avoid any inadvertent unblinding. Primary MR and SPECT analyses in CE‐MARC used all data from the multicomponent assessments, but individual components such as perfusion were also scored separately. MR perfusion image quality was graded subjectively on a scale of 1–4 as follows: 1 = unusable; 2 = poor quality; 3 = adequate; 4 = high quality. Rest and stress perfusion were then evaluated visually for 16 of 17 AHA/ACC model segments^12^ (excluding the apical cap), as 0 (normal), 1 (equivocal), 2 (subendocardial ischemia), or 3 (transmural ischemia). SPECT perfusion imaging was graded in 17 segments on a scale of 0–4 as follows: 0 = normal uptake; 1 = equivocal; 2 = moderately reduced; 3 = severely reduced; 4 = absent for both rest and stress. An overall assessment of myocardial perfusion in each patient as "normal," "probably normal," "probably abnormal," and "abnormal" was made using both rest and stress images, independently for MR and SPECT.

### Hemodynamics

Hemodynamic response to adenosine was assessed by comparing hemodynamics at rest and after 2–3 minutes of adenosine infusion, immediately prior to contrast administration. An inadequate response was defined as SBP decrease <10 mmHg or heart rate increase <10 beats/min with adenosine infusion.^13^

### Coronary Angiography

Radiographic angiographic QCA findings were taken from the original blinded CE‐MARC analysis, performed offline using QCAPlus software (Sanders Data Systems, Palo Alto, CA). As well as recording degree of luminal stenosis per segment, we calculated the Duke jeopardy score to estimate the myocardium at risk.^11^ To calculate this score, the coronary tree was divided into six segments (left anterior descending artery, major septal perforator, major diagonal branch, circumflex artery, major obtuse marginal branch artery, and posterior descending artery). Two points were given for each segment that would be compromised by a significant stenosis, giving a total maximal score of 12 per patient.

### Myocardial Perfusion Reserve

Independent of the multivariate analysis, MPR in the 35 patients with false‐negative perfusion MR studies was calculated. MPR estimation was performed offline using PMI v. 0.4 software.^14^ Deconvolution was performed on MR stress and rest perfusion images using a Fermi model applied to the first pass,^15^, ^16^ with arterial input defined in the LV blood pool, and the whole mid‐LV short axis myocardial slice as tissue response. Regions of interest were carefully selected by an experienced reader (4 year' experience) to be as large as possible but to avoid dark rim artifact, trabeculations, and papillary muscles, and manually corrected to account for respiratory motion. MPR was calculated by dividing stress blood flow by the rest value. For comparison, MPR was also calculated in 20 randomly selected CE‐MARC patients with normal x‐ray coronary angiography and compared to the false‐negative group.

### Statistical Analysis

Statistical analysis was performed using SAS 9.2 (SAS Institute, Cary, NC). The four prespecified potential factors (poor quality stress perfusion MR images, triple‐vessel disease, inadequate hemodynamic response, and Duke jeopardy score), were assessed individually using binary logistic regression, modeling false‐negative status in a univariate manner. A multivariate model was fitted to the data in which these factors were included. To minimize errors related to multiple significance testing, no model‐building strategy was followed. Odds ratios (OR) for a false‐negative rather than a true‐positive result and *P*‐values for differences between estimated OR and 1 were calculated for all models. OR is denoted with 95% confidence intervals (CIs) in parentheses. For the Duke jeopardy score, OR was estimated for the minimum 2‐point increase in score. For categorical variables, OR was estimated relative to the absence of the factor in question. A complete‐case analysis was performed in the multivariate regression analysis: four patients (1.5%, all true‐positive) with were excluded due to incomplete data on one or more factors. All other patients had complete data on all factors. Normality for MPR data was tested using the Shapiro–Wilk test; false‐negative and control MPR data were compared using Student's *t*‐test. Severity of significant stenosis was not regarded as normally distributed and compared using the Wilcoxon rank‐sum test.

## Results

Of the 752 patients enrolled into CE‐MARC, 676 completed both MR perfusion and x‐ray angiography; of these, 266 (39%) patients had clinically significant angiographic CHD. One patient had perfusion image quality recorded as “unusable,” and was excluded from further analysis, which left 265 patients for the current analysis. The population of false‐negative patients was 35 (5% of the CE‐MARC population), all of whom had MR perfusion graded as “probably normal” (rather than “normal”). Overall, 230/752 (34%) patients had true‐positive findings. No false‐negative patients had myocardial scar visible on late gadolinium enhancement MR. Patient characteristics are shown in Table 1.

**Table 1:** Baseline Characteristics for False‐Negative and True‐Positive Perfusion MR

	False‐negative patients	True‐positive patients
*n* (%)	35 (13%)	230 (87%)
Age (years)	61 ± 7	62 ± 9
Male	29 (83%)	180 (78%)
Body‐mass index (kg/m^2^)	28.3 ± 4.0	29.0 ± 4.0
Resting BP (mmHg)	125/71 ± 20/10	142/79 ± 21/11
Hypertension	17 (49%)	122 (53%)
Current smoker	5 (14%)	42 (18%)
Total cholesterol	5.3 ± 1.2	5.3 ± 1.3
Diabetes mellitus	3 (9%)	35 (15%)
Framingham risk	14.4 ± 2.6	14.7 ± 3.0
LAD disease	19 (54%)	149 (65%)
Circumflex disease	16 (46%)	110 (48%)
RCA disease	8 (23%)	97 (42%)
Left main disease	2 (6%)	20 (9%)
Stenosis severity	80% ± 8	86% ± 11
Single vessel disease	28 (80%)	115 (50%)
Double vessel disease	6 (17%)	77 (33%)
Triple‐vessel disease	2 (6%)	38 (17%)

Data as *n* (%) or mean ± SD.

### Overall Assessment

**Figure 1:**
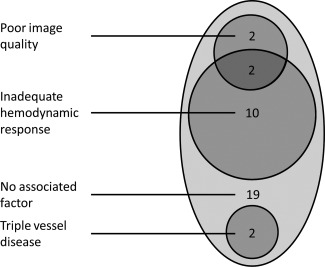
Candidate factors associated with a false‐negative MR perfusion scan (*n* = 35).

Sixteen of the 35 (46%) false‐negative patients had one or more of the prespecified candidate factors under investigation (Fig. 1). Two patients had more than one factor.

### Individual Factor Analysis

Individual patients are summarized in Table 2. Four (11%) MR perfusion studies in the false‐negative group and five (2%) studies in the true‐positive group were graded as “poor quality” (multivariate OR 4.14 (95% CI 0.82–20.96), *P* = 0.09, Table 3).

**Table 2:** Individual Patient Characteristics and Associated Factors

Patient	Age	Poor MR image quality	Inadequate hemodynamic response	Triple‐vessel disease	Duke jeopardy score	Maximum angiographic severity	SPECT normal or probably normal
1	48	No	No	No	2	71	Yes
2	71	No	Yes	No	2	84	No
3	61	No	Yes	No	2	84	No
4	61	No	No	No	2	92	Yes
5	59	No	No	No	2	92	Yes
6	51	No	No	No	2	74	No
7	65	No	No	No	2	71	Yes
8	60	No	Yes	No	0	91	No
9	62	No	No	No	4	78	No
10	50	No	No	No	2	78	Yes
11	58	No	Yes	No	4	89	Yes
12	55	No	No	Yes	6	85	Yes
13	50	No	Yes	No	2	99	No
14	72	Yes	No	No	0	88	Yes
15	53	No	No	No	4	79	No
16	67	No	No	No	0	78	No
17	68	No	No	No	2	84	Yes
18	73	No	No	No	2	70	No
19	69	Yes	No	No	4	90	Yes
20	57	No	Yes	No	4	98	No
21	61	No	Yes	No	4	82	Yes
22	70	No	Yes	No	2	71	No
23	66	No	No	No	2	82	Yes
24	61	No	No	No	4	88	Yes
25	59	Yes	Yes	No	0	94	No
26	65	No	No	No	4	76	Yes
27	55	No	No	No	4	74	Yes
28	57	No	No	No	2	75	Yes
29	66	No	No	No	2	85	Yes
30	63	No	No	No	4	89	No
31	69	No	Yes	No	2	89	Yes
32	64	No	No	Yes	8	71	No
33	71	No	No	No	0	83	Yes
34	50	Yes	Yes	No	2	74	Yes
35	57	No	Yes	No	2	79	Yes

**Table 3:** Univariate and Multivariate Regression Analyses

Variable	False‐negative *n* (%)	True‐positive *n* (%)	Odds ratio (95% confidence interval)		*P* value	
			Univariate	Multivariate	Univariate	Multivariate
Poor MR image quality	4 (11%)	5 (2%)	5.75 (1.47–22.59)	4.14 (0.82–20.96)	0.0121	0.0861
Inadequate hemodynamic response	12 (34%)	51 (22%)	1.79 (0.83–3.85)	1.57 (0.65–3.76)	0.1354	0.3140
Triple‐vessel disease	2 (6%)	38 (17%)	0.31 (0.07–1.33)	1.19 (0.20–7.14)	0.1144	0.8498
Duke jeopardy score (2 point increment)	2.6 ± 1.7	5.4 ± 3.0	0.34 (0.21–0.53)	0.35 (0.22–0.55)	<0.0001	<0.0001

Odds ratios are for a patient with CAD having a false‐negative MR perfusion result. A value >1 indicates increased likelihood of a false‐negative (rather than true‐positive) MR study in the population with the variable indicated or as a result of a 2‐point increase in Duke score.

Twelve false‐negative studies (34%) had inadequate hemodynamic response to adenosine, of which two also had poor image quality. Six (50%) of these 12 patients were positive for inducible ischemia on SPECT imaging. Ten (83%) of these 12 patients also had inadequate response at the time of SPECT. Fifty‐one (22%) true‐positive studies fulfilled the criteria for inadequate hemodynamic response (multivariate OR 1.57 (0.65–3.85), *P* = 0.3).

MPR in false‐negative patients was 1.9 ± 0.8 and in controls was 2.1 ± 0.5 (*P* = 0.3). One (3%) false‐negative patient had MPR more than 2 SD below controls. MPR values in both false‐negative patients with triple vessel disease were 1.7 and 2.1, lower than normal but still indicating some increase in myocardial blood flow with adenosine.

Twenty‐eight of 35 (80%) of false‐negative MR studies had angiographic single vessel disease, as compared to 115 (50%) of true‐positive patients. Two (6% of 35) false‐negative MR studies had triple‐vessel disease, as compared to 38 (17% of 265) of true‐positive studies (multivariate OR 1.19 (0.20–7.14), *P* = 0.8). Mean severity of significant stenosis in false‐negative patients was 80% ± 8 and 86% ± 11 in true‐positive patients (*P* < 0.001). The mean Duke jeopardy score was 2.6 ± 1.7 for the false‐negative studies, significantly lower than the score for true‐positive studies, 5.4 ± 3.0 (multivariate OR 0.35 (0.22–0.55, *P* < 0.0001 for a 2‐point difference).

**Figure 2:**
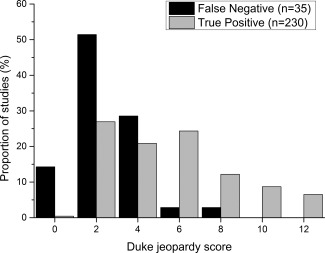
Distribution of Duke jeopardy scores in false‐negative and true‐positive studies.

**Figure 3:**
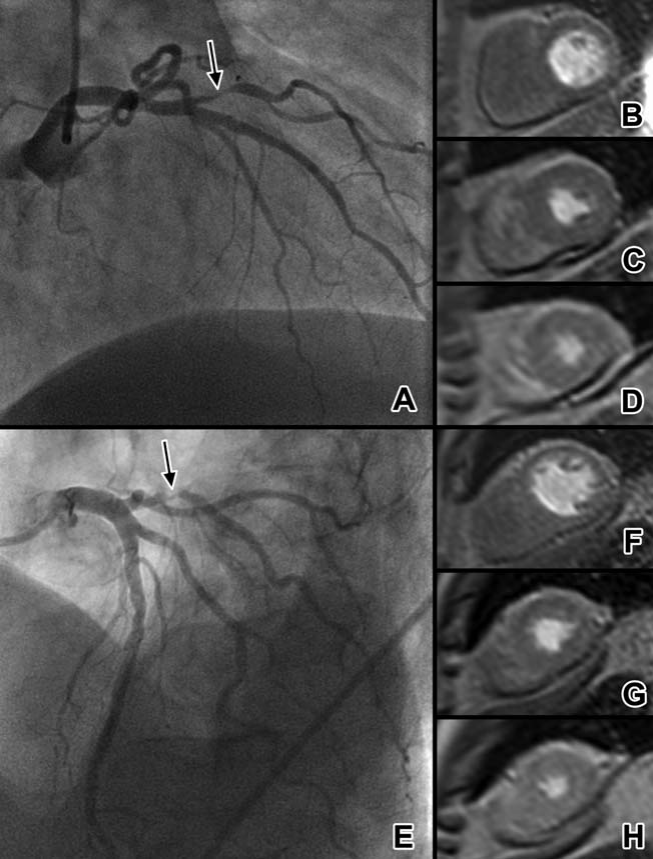
Example of two typical patients with false‐negative MR perfusion. A patient with diagonal branch stenosis (**A,** arrowed) and absence of a clear perfusion defect on basal **(B)**, mid‐ventricular **(C)**, and apical **(D)** first‐pass perfusion MR images. SPECT imaging showed a subtle anterolateral perfusion defect. Duke jeopardy score was 2. Another patient with obtuse marginal branch stenosis (**E,** arrowed) again has no visible perfusion defect on MR **(F–H)**. SPECT was negative in this case, with Duke jeopardy score of 2. MR appears to more commonly miss stenoses which subtend relatively small areas of myocardium.

Figure 2 shows the distribution of Duke jeopardy scores. Figure 3 shows images from two typical patients in the false‐negative population. SPECT was reported as “normal” or “probably normal” in 21 (60%) of false‐negative and 63 (27%) of true‐positive MR perfusion studies (univariate OR 0.2 (0.10–0.47), *P* < 0.0001). Fourteen (50%) of the 28 false‐negative MR studies with single‐vessel disease also had a negative SPECT study. Of 19 false‐negative MR studies with adequate hemodynamic response, diagnostic image quality and no other associated factors, mean QCA diameter of angiographic stenosis was 79 ± 7%, the Duke score was 2.4 ± 1.3, and SPECT was reported as “normal” or “probably normal” in 13 (68%).

### Multivariate Analysis

From the univariate analysis, poor quality image and Duke jeopardy score were significantly related to a false‐negative MR perfusion result. In a multivariate logistic regression model, the association between poor image quality and false‐negative findings was not statistically significant (Table 3). The reduced likelihood of a false‐negative finding in patients with a higher Duke jeopardy score remained strongly statistically significant.

## Discussion

We explored factors associated with a false‐negative cardiovascular MR perfusion scan from the CE‐MARC study, which provides the currently largest prospective real‐world evaluation of MRI in CHD. The main finding was that false‐negative MR perfusion results were associated with low volumes of myocardium at risk. Triple‐vessel disease and hemodynamic response to adenosine did not significantly influence the likelihood of a false‐negative study. All false‐negative cases received MR perfusion scoring with low observer confidence. SPECT was normal in 60% of all false‐negatives, suggesting that QCA, which was the reference standard in CE‐MARC, may have overestimated the functional significance of visual angiographic stenosis in many patients.

Despite a large body of evidence for the accuracy of MR stress perfusion accrued over the past 20 years, there are few dedicated evaluations of the predictors and relative incidences of false‐negative scans. Stress myocardial perfusion MR requires high temporal and spatial resolution across all coronary territories, and must accurately image patients with high heart rates and sometimes suboptimal breath holding. Despite challenges, false‐negative MR perfusion studies are uncommon. A recent meta‐analysis of perfusion MR revealed false‐negative rates of 0–16% from the 34 vasodilator stress studies analyzed, comparable to our incidence of 5% of all CE‐MARC patients.^6^ The largest study in the meta‐analysis (21 false‐negative patients) was prone to verification bias, as it was neither randomized nor prospective.^17^ In contrast to other studies that have not performed reference angiography in cases of negative noninvasive imaging, the CE‐MARC study is well placed to robustly evaluate false‐negative rates, as it avoids referral or verification bias because all patients were scheduled to have MR, SPECT, and radiographic angiography, regardless of the noninvasive imaging results.

In CE‐MARC, just 4 (11%) of the false‐negative MR perfusion studies had image quality graded as "poor." While having a significant univariate relationship to a false‐negative finding, this was not the case when adjusting for other variables. In the meta‐analysis described above,^6^ there was no significant chronological change in false‐negative rates, suggesting that improvements in imaging technique, while striving to improve diagnostic accuracy, may have a limited role in reducing the frequency of false‐negative results. Consistent with this notion, we found that most false‐negative results were associated with factors outside of the imaging acquisition.

Other reasons for false‐negative MR perfusion studies have been proposed. False‐negative perfusion imaging may occur in the context of multivessel disease, which may give rise to "balanced ischemia."^18^, ^19^ A small proportion of false‐negative studies in CE‐MARC had significant triple‐vessel disease, with no significant difference in likelihood of triple‐vessel disease in the false‐negative and true‐positive groups. These data support the robustness of MR perfusion in the detection of significant CHD in the context of multivessel disease. In addition, the lateral and posterior walls are said to be more difficult to image robustly, but there was no predominance of false‐negative patients with circumflex coronary artery disease in the current analysis.

Our data suggest that smaller areas of ischemia are associated with false‐negative studies. The Duke jeopardy score is an estimate of myocardium‐at‐risk based on radiographic angiography,^11^ with a maximum score of 12. Low Duke jeopardy score (ie, less myocardium at risk) had the strongest association with false‐negative MR studies of the variables tested. Duke score is known to show strong correlation with functional significance of coronary stenoses by FFR.^20^ The mean Duke score for false‐negative MR studies indicated relatively low amounts of myocardium at risk, which is associated with an excellent 5‐year survival of 95–97%.^21^ Furthermore, the majority of false‐negative studies had angiographic single‐vessel disease.

The discrepancy between angiographic stenosis severity and functional flow attenuation that may lead to a perfusion defect is well known.^22^ In CE‐MARC, concordance of negative SPECT and MR perfusion studies was seen in 60% of patients. In the current subanalysis, the 19 patients who did not have an alternative associated factor had a low mean jeopardy score and the majority had a negative SPECT result, despite a high mean angiographic stenosis severity. These results support the notion that in a substantial proportion of false‐negative MR studies, QCA angiographic severity of stenosis overstates its hemodynamic significance.

A lack of response to adenosine has been hypothesized to result in false‐negative studies through reduced flow attenuation to myocardium subtended by stenotic arteries. The standard dose of 140 μg/kg/min given in CE‐MARC and the vast majority of other studies has been found to cause an inadequate hemodynamic response in 16–18% of patients.^13^, ^23^ In the current subanalysis, rates of inadequate hemodynamic response were numerically higher in the false‐negative studies, but without statistically significant difference to the true‐positive studies. Furthermore, 31% of false‐negative MR studies with inadequate hemodynamic response had inducible ischemia on SPECT, which used the same adenosine protocol. Myocardial perfusion reserve, the ratio of hyperemic to resting myocardial blood flow,^15^ was similar between false‐negative studies and normal controls. These results all suggest that adenosine response was similar in the two groups.

Despite the large population of CE‐MARC, relatively few patients had false‐negative MR perfusion studies, reflecting the comparatively high sensitivity of perfusion MR. Inclusion criteria may be related to false‐negative rates; the CE‐MARC study included patients with stable symptoms thought to be angina and at least one cardiovascular risk factor. The sample size limited the number of possible factors that could be meaningfully analyzed in a multivariate model to the four prespecified factors of interest. CE‐MARC, as a pragmatic study reflecting real‐world practice and in keeping with its study design in 2006 (prior to publication of the FAME study^24^), did not routinely perform catheter‐based fractional flow reserve (FFR) measurements to address the functional consequence of angiographic stenoses. Given the high sensitivity of MR, the risk of performing FFR in all negative studies would be difficult to justify. The corroboration between MR and SPECT, both of which were negative in the majority of unexplained false‐negative cases, provides evidence to this end. We did not attempt to correlate hypoperfused territories with coronary anatomy, as subanalysis of this relatively small population was felt to be impractical, and of limited pragmatic use. As per trial design, image interpretation was performed by consensus of two experienced readers. Image quality ratings have a subjective component and may be influenced by the overall image capabilities of the pulse sequence. The CE‐MARC perfusion method was not optimized for quantitative analysis and the accuracy of the MPR measurements may be limited.

In conclusion, of 676 patients with cardiovascular MR and coronary angiography in the CE‐MARC study, the incidence of false‐negative MR perfusion studies was 5%. False‐negative MR perfusion is significantly more likely to occur in patients with low Duke jeopardy score and hence good prognosis. The adequacy of adenosine response by hemodynamic measures was similar between false‐negative and true‐positive groups. Multivessel disease and low image quality scores were not significantly associated with false‐negative MR perfusion.
